# Supplementary material for: The Clinical and Medico-Legal Aspects in the Challenge of Transfusion-Free Organ Transplants: A Scoping Review
Source: J Clin Med. 2025 Aug 1;14(15):5444. doi: 10.3390/jcm14155444 (PMC12347238; doi:10.3390/jcm14155444)

1. **Prisma flowchart for kidney transplants**

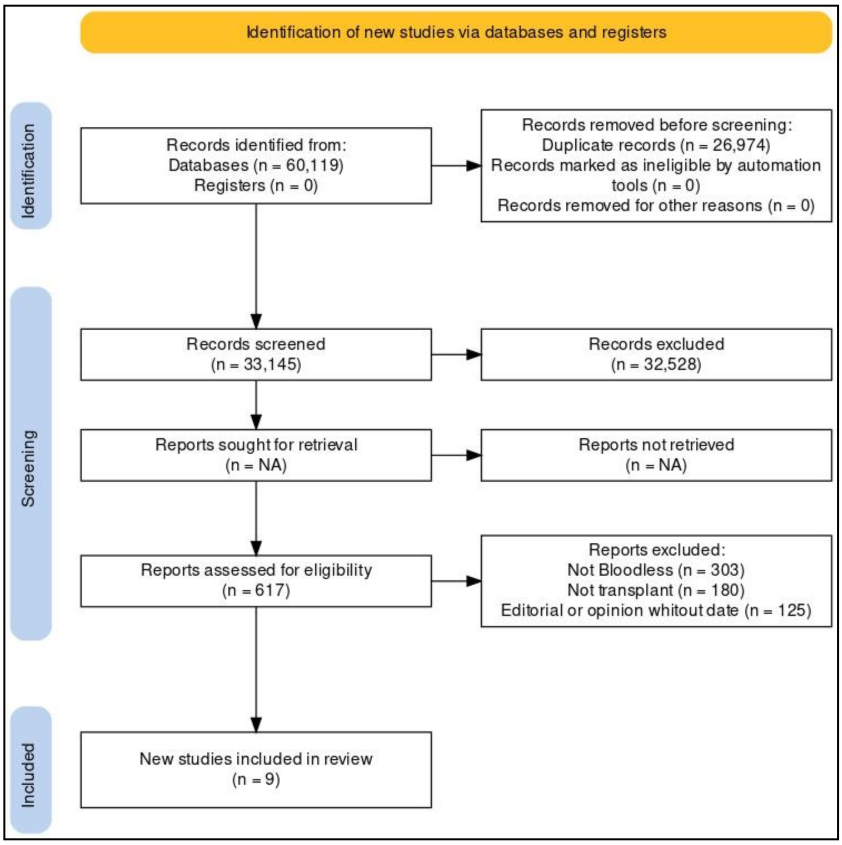

2. **Prisma flowchart for liver transplants**

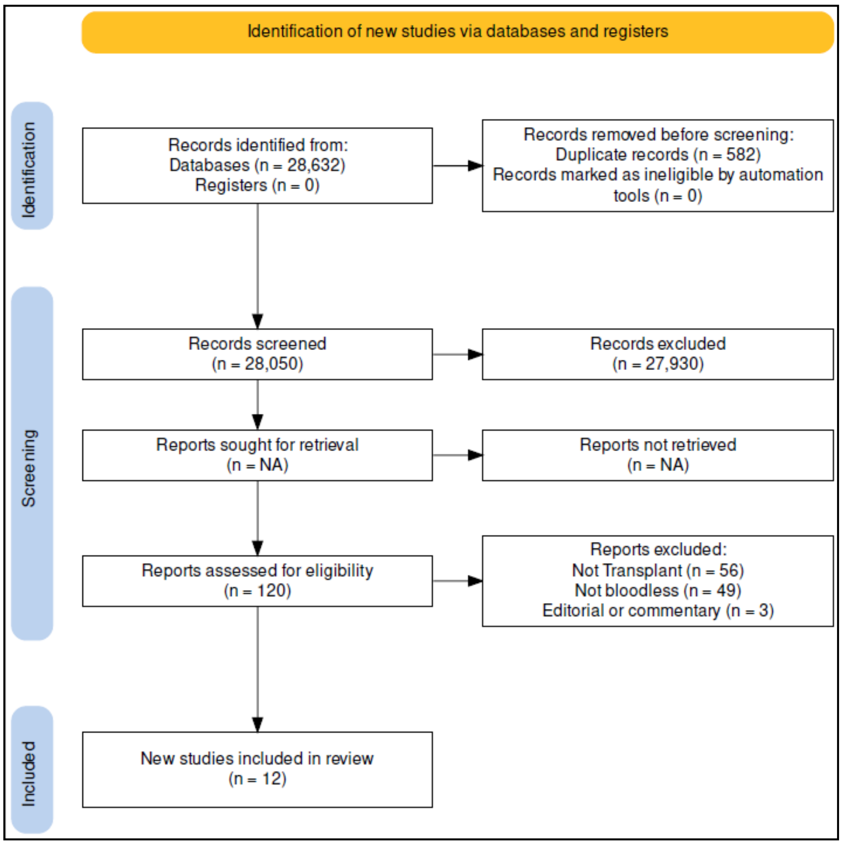

3. Prisma flowchart for hearth transplants

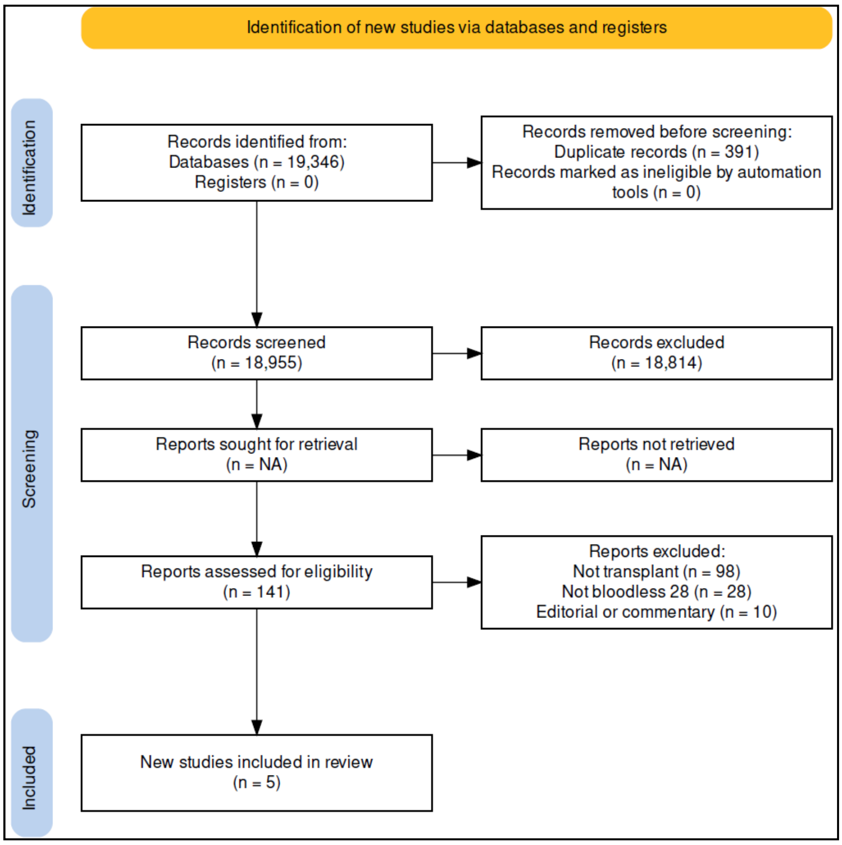

4. Prisma flowchart for lung transplants

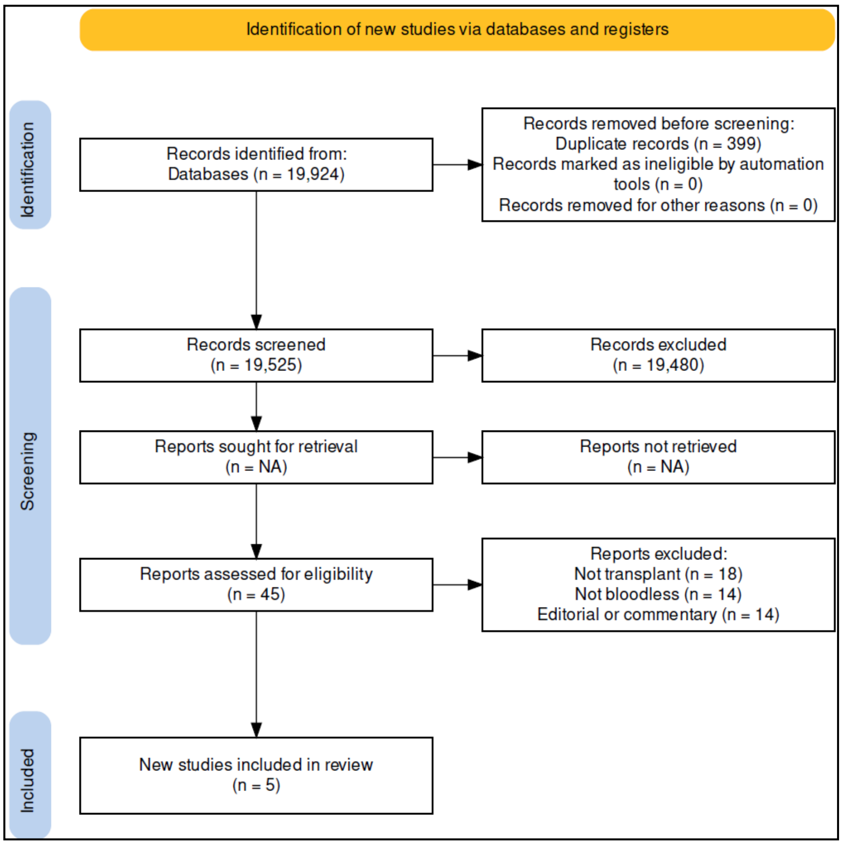

Supplement: Supplementary file 1 [file jcm-14-05444-s001.zip › jcm-3667849 Supplementary file 1 PRISMA flowchart.pdf]
